# A Brief Overview of Recent Pediatric Physical Therapy Practices and Their Importance

**DOI:** 10.7759/cureus.47863

**Published:** 2023-10-28

**Authors:** Chavan Srushti Sudhir, H V Sharath

**Affiliations:** 1 Department of Paedatric Physiotherapy, Ravi Nair Physiotherapy College, Datta Meghe Institute of Higher Education & Research (Deemed to be University), Wardha, IND; 2 Department of Paediatric Physiotherapy, Ravi Nair Physiotherapy College, Datta Meghe Institute of Higher Education & Research (Deemed to be University), Wardha, IND

**Keywords:** infant conditions, autism, preterm infants, cardiorespiratory conditions, paediatric physiotherapy

## Abstract

Recent years have seen a substantial increase in interest in pediatric physical therapy, which is a reflection of improvements in its methods and the rising understanding of its significance in child development and rehabilitation. This review provides a concise overview of the latest trends and techniques in pediatric physical therapy, emphasizing the integration of innovative technologies, evidence-based interventions, and holistic approaches. For children with varied developmental, congenital, and acquired disorders, the significance of early intervention and individualized treatment programs is emphasized, underlining the important influence of prompt interventions on long-term functional results and quality of life. To guarantee comprehensive and coordinated care, the study also examines the interdisciplinary character of pediatric physical therapy placing special emphasis on collaboration with families, caregivers, educators, and healthcare professionals. It also emphasizes the importance of continuing research, instruction, and lobbying to improve the effectiveness and availability of pediatric physical therapy services, eventually promoting the overall well-being of kids and their families.

## Introduction and background

Pediatric physical therapy is a branch of physiotherapy that helps treat or develop movement issues in infants and young children and enhances a child's developmental stages. Orthopedics, congenital malformations, neurology, neuropsychiatry, respiration, and preterm are among the systems that affect children [1]. Pediatric physical therapists address developmental delay and neuromotor disorders. They treat children of all ages, from infants to teenagers, and they have particular knowledge and training in growth and development, syndromes, and diagnosis (Table 1) [2].

**Table 1:** Some important treatment approaches used by pediatric physical therapists

Techniques	Description
Bobath approaches	This neurodevelopmental therapy is utilized to improve specific developmental stages, postural deformity or aberrant patterns, as well as to improve sensory input.
Motor learning	Infants with cerebral palsy typically benefit from this practice.
The Margaret Rood approach	This approach uses both superficial and deep stimulation to stimulate motor activity.
The Doman-Delecto technique	It uses steady, repeating motions of patterns of natural sequential development movement to stimulate the brain's dormant central nervous system pathways.
The Kabat-Knott-Voss technique	It uses practical movement patterns to facilitate proprioceptive neuromuscular function.

The pediatric physical therapist may collaborate with a wide range of specialists due to the complicated requirements of the child and the family, including teams from medicine, nursing, social work, education, and psychiatry, as well as speech and occupational therapists [3,4]. Even though India is still in its infancy, the breadth has significantly expanded in the last several years to decades. Despite this point of view, there is controversy around using therapeutic touch in pediatric physical therapy, particularly for children with cerebral palsy [5,6]. Physiotherapists who work with children are experts in evaluating, identifying, diagnosing, and treating movement abnormalities and physiological conditions (Table 2). They treat children (infants up to 19 years) in the areas of orthopedics, congenital anomalies, neurology, neuropsychiatry, breathing, and preterm [7-9]. 

**Table 2:** List of some important conditions that we are going to review ASD: autism spectrum disorder; ADHD: attention-deficit hyperactivity disorder; ARDS: acute respiratory distress syndrome.

Conditions	Definition
Preterm infants	Babies born alive before 37 weeks of pregnancy are considered preterm. Based on the gestational age, preterm birth is divided into the following subcategories: very premature birth (less than 28 weeks), extremely preterm (28 to under 32 weeks), and late to moderate preterm (32-37 weeks).
Cerebral palsy	Mobility, balance, and posture are all impacted by a set of illnesses collectively referred to as cerebral palsy. During early childhood or preschool, signs and symptoms first arise. Poor mobility brought on by cerebral palsy is indicated by abnormal posture, floppiness or spasticity of the limbs and trunk, excessive reflexes, uncontrollable motions, shaky walking, or any other combination of these symptoms.
Torticollis	It is a condition that causes a baby's head to twist and tilt to one side because of the neck muscles. Torticollis is also known by the term "WRYNECK." Torticollis can be caused by a variety of conditions, including muscular fibrosis, congenital problems with the spine, and toxic or traumatic brain injury. Congenital cases of torticollis and acquired cases can be broadly classified.
Spina bifida	It is a spinal condition that is typically noticeable from birth. This specific abnormality affects the neural tube. Spina bifida can appear anywhere along the spine if the neural tube does not completely close. Spina bifida, myelomeningocele, and meningocele are the types of spina bifida.
ASD	Autism spectrum disorder is defined by limited repetitive behavioral patterns and trouble with social communication. The term "spectrum" describes the wide range of signs and degrees of severity connected with autism spectrum disorder.
Down syndrome	It has a certain facial appearance, a developmental delay, and an intellectual handicap. It could be related to thyroid or heart problems. Usually, a "nondisjunction" flaw in cell division causes it. Down syndrome is a hereditary disorder caused by a faulty cell division process that adds an extra whole or partial copy of chromosome 21. This excess genetic material contributes to the physical characteristics and developmental problems of Down syndrome.
ADHD	ADHD is the most common neurobehavioral illness in children. It frequently receives its initial diagnosis as a youngster and persists into adulthood. It is well established that ADHD can have negative consequences on a person's academic performance, career prospects, and even day-to-day functioning. ADHD is thought to be a serious and persistent disorder.
Erb's palsy	An arm paralysis caused by injury to the upper group of the arm's major nerves, particularly the upper trunk C5-C8 nerves. The brachial plexus is composed of the ventral rami of the thoracic nerve T1 and the spinal nerves C5-C8. The majority of the time, but not always, shoulder dystocia following a difficult delivery is to blame for these injuries. Depending on the type of lesion, the paralysis could go away on its own with time, call for therapy or surgery.
ARDS	This condition causes fluid to accumulate in the air sacs of the lungs, preventing oxygen from reaching vital organs. It affects infants as a result of an accident or infection. It can be used to diagnose infants with hypoxia.

## Review

Study selection

**Figure 1:**
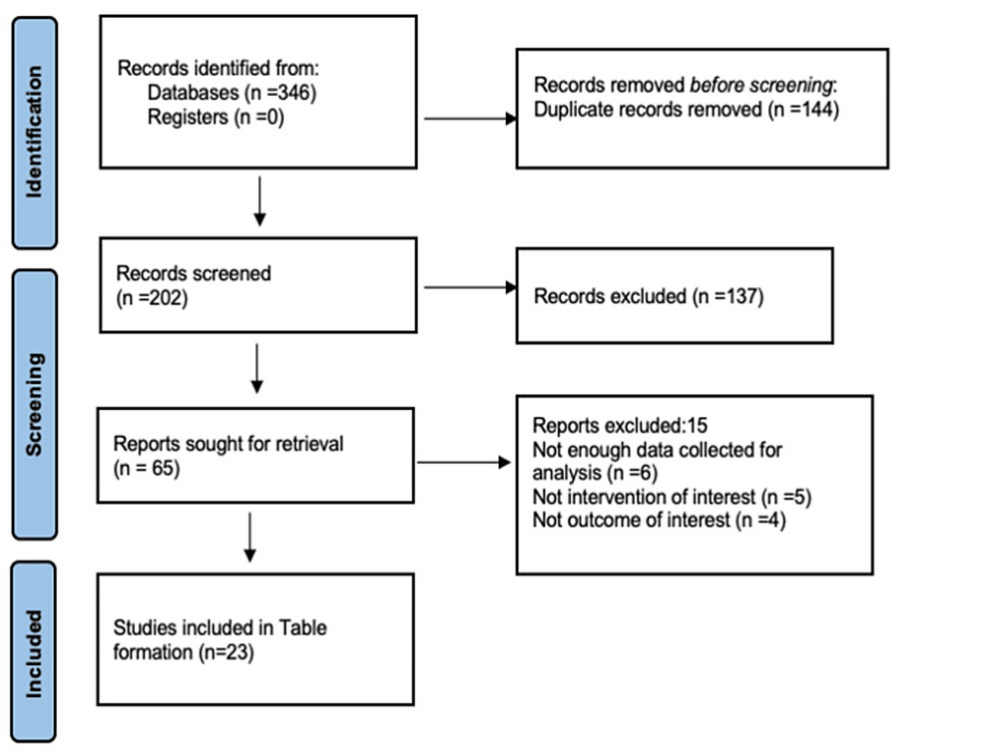
PRISMA flow diagram for reviews PRISMA, Preferred Reporting Items for Systematic Reviews and Meta-Analyses.

We included all research articles like randomized and non-randomized clinical studies, systematic reviews, and experimental studies of recent pediatric physical practices over the past five years; the English-language literature was searched on Google Scholar, Medline, Cochrane Library, and PubMed. Between 2019 and March 2023, 346 articles were searched using the terms paediatric physiotherapy, modern treatment practices, and advanced physiotherapy. Twenty-three papers were included in the 50 articles eligible for full-text review out of the 346 articles (Figure 1), which displays a database search, and data extraction demonstrates the outcome of the article selection process. A list of the examined papers is shown in Table 3, which provides a quick summary of current pediatric physical procedures.

**Table 3:** A summary of the articles reviewed for recent pediatric physical practices and its importance GMA: General Movement Assessment; AIMS: Alberta Infant Motor Scale; NDD: Neurodevelopmental Delay; IH: Intermittent Hypoxia; LRT: Lower Respiratory Tract; NDT-B: Neurodevelopmental Therapy Method-Bobath; CMT: Congenital Muscular Torticollis; NTDs: Neural Tube Defects; SB: Spina Bifida; SSC: Stretch Shortening Cycle; DS: Down Syndrome; ADHD: Attention-Deficit Hyperactivity Disorder; HCs: Healthy Asymptomatic Controls; MOOSE: Meta-analysis of Observational Studies in Epidemiology; NBPP: Newborn Brachial Plexus Palsy.

S. No.	Author name/year of publication	Aim of the study	Methodology	Conclusion
1.	Embarek Hernandez M et al. 2022 [8]	To evaluate the evidence about the effect of a multisensory stimulation program applied on preterm infants, compared to any intervention or standard case.	The data sources were Medline (PubMed, EBSCO), Scopus, and Web of Science. The analysis looked at randomized clinical studies published between 2015 and 2021 that looked at multimodal stimulation therapy in preterm infants born less than 37 weeks of pregnancy.	Preterm newborns may benefit from multisensory stimulation in terms of eating, psychomotor development, and visual function.
2.	Canan Yildirim et al. 2022 [9]	To compare the GMA with the AIMS for the identification of cerebral palsy (CP) and NDD in preterm babies.	There were 75 preterm infants between 24 and 37 weeks of gestation. The AIMS was used to assess the infants throughout their periods of writhing, fidgeting, and between 6 and 12 months of corrected age, respectively.	Cerebral palsy and neurodevelopmental delay can be predicted using the general movement assessment technique. Combining the Alberta Infant Motor Scale and overall Movement Optimality Score may help clinicians make a precise and accurate diagnosis of cerebral palsy and neurodevelopmental delay.
3.	Stephanie Margarete Mueller et al. 2023 [10]	Evaluation of the effect of parental and direct medical interventions on the incidence of IH in extremely preterm neonates is the study's main objective.	It was conducted using an observational design with intra-individual comparisons. Time-lapse video and the blood oxygen saturation level [spO_2_] were both recorded. IH occurrence frequency, duration, and time were also recorded.	Future studies should examine the ways in which preterm infants understand touch. The basic touch triggers of intermittent hypoxemia, and the best handling strategies for preventing intermittent hypoxemia during direct medical care.
4.	Hege H Clemm et al. 2018 [11]	The purpose of this review is to give an overview of what is currently known regarding bronchial hyperresponsiveness in this susceptible group of persons.	Among the techniques used in microbiology are the antigen test, polymerase chain reaction, direct immune-fluorescence, and [paired] serology. Naso-oropharyngeal swabs from the upper respiratory tract are frequently employed as stand-in markers for the LRT.	Although lung illness following preterm birth and respiratory symptoms in asthma may resemble one another, clinical experience and research demonstrate that preterm delivery symptoms are indicative of a different disease entity.
5.	Merete Aarsland Fosdahl et al. 2019 [12]	To examine the impact of progressive resistance training and stretching on the range of motion and muscle strength in kids with cerebral palsy.	Stretching the hamstrings for 16 weeks, followed by 16 weeks of maintenance exercises that target the lower extremities.	After a 16-week stretching and progressive resistance training program, followed by a 16-week maintenance program, the intervention group showed no significant improvements in passive, active popliteal angle, and muscle strength.
6.	Mohamed Ali Elshafey 2022 [13]	The purpose of the study is to evaluate the effects of a core stability training program on balance, coordination, and the degree of ataxia in children with cerebellar ataxic cerebral palsy (CP).	The split groups were analyzed before and after therapy using the Bruininks-Oseretsky test of motor competence, the scale for assessment and rating of ataxic, the balance error scoring system scale, and the Humac balancing system scores.	When combined with a conventional physical treatment routine, the core stability program helps children with cerebellar ataxic cerebral palsy improve their balance and coordination.
7.	Gonul Acar et al. 2022 [14]	The capacity to feed and swallow was impacted by the structured NDT-B approach in patients with cerebral palsy (CP) and eating disorders.	Bobath approach affected individuals with cerebral palsy and eating difficulties during their feeding and swallowing activities.	Plans for treatment should include neck and trunk stability exercises based on the Bobath neural tube dysfunction..
8.	Bax et al. 2005 [15]	To assess how training in both proprioceptive and visual skills at the same time affects the gait characteristics in kids with spastic diplegic cerebral palsy.	Using Tekscan's walk way pressure system, the gait parameters of 30 spastic diplegic children (ages 4-6) were assessed before and after treatment.	Proprioceptive and visual training can be beneficial for children with spastic diplegic cerebral palsy, although it has little impact on kinetic gait characteristics.
9.	Hilal Keklicek 2018 [16]	To determine the effectiveness of soft tissue mobilization in treating congenital muscle torticollis in infants with mild to moderate head tilt.	29 infants with congenital muscle torticollis between the ages of 0 and 9 months and head tilts between 50 and 200 were divided into two groups. Both groups received a foundational home program that included alignment, handling techniques, stretching, and strengthening exercises.	Approaches for soft tissue mobilization are effective in treating CMT and yielding favorable effects more quickly.
10.	Irene Cabrera Martos et al. 2015 [17]	To investigate torticollis (congenital or acquired) on the attainment of particular gross motor milestones in infants with plagiocephaly.	175 newborns with plagiocephaly, with or without torticollis, had baseline measurements taken for anthropometric and clinical factors.	The results show that in infants with plagiocephaly, congenital or acquired torticollis is a significant factor influencing gross motor development.
11.	Castilla et al. 2023 [18]	To investigate recent research on physical therapy (PT) diagnosis, prognosis, and treatment of congenital muscular torticollis to update the evidence-based clinical practice guideline for PT care of congenital muscular torticollis.	In order to find studies that influenced physical therapy diagnoses, prognoses, or therapies for newborns and kids with congenital muscle torticollis, seven databases were searched from 2012 to 2017.	According to recent studies, breech presentation, low birth weight, and motor asymmetry are all risk factors for prolonged treatment times. The scientific basis for microcurrent therapy is growing.
12.	Laura Avagliano et al. 2019 [19]	The aim of the current study is an overview of neural tube defects.	In order to provide crucial information based on the classification of neural tube abnormalities for clinical and scientific use, a review of the literature on histological and pathological features, epidemiology, prenatal diagnosis, and prognosis depending on the type of defect was conducted.	NTDs are the second most frequent congenital anomaly that affects how the human central nervous system develops.
13.	Rocque et al. 2021 [20]	To list the factors influencing self-management behaviors, the development of general or condition-specific self-management measures used with the spina bifida population, and the development and/or outcomes of interventions to improve self-management in SB.	Self-management encompasses self-management techniques, preliminary information on spina bifida-specific instruments, factors that must be considered during the creation and testing of future interventions, and knowledge gaps.	The state of the science of self-management, self-management behaviors, prospective spina bifida instruments, considerations for developing and evaluating potential future therapies, and gaps in the literature are all outlined in this paper.
14.	Holly Hodges 2020 [21]	The aim of the study is to have an overview of autism spectrum disorder	The authors examine the data supporting the hypothesis that autism spectrum disorder is a neurobiological condition influenced by both hereditary and environmental variables that affect the developing brain and list risk factors for the condition.	No clear causal pathway has been established, despite the fact that research is still uncovering traits that are associated with the risk of autism spectrum disease and that these findings may direct future etiologic exploration.
15.	Nora Shields 2021 [22]	T o show the preliminary evidence that exercise has positive short-term effects on cognitive touch	The most recent studies have examined how aerobic exercise affects both cognitive function and low-grade systemic inflammation.	Depending on the stage of life, a physiotherapist's role when working with a person with Down syndrome generally includes encouraging physical activity.
16.	A R Azab et al. 2022 [23]	To determine how a three-month SSC program on a trampoline affected the children with DS in terms of muscle strength and postural control.	Both before and after therapy, lower limb muscle strength and postural stability (including the anterior/posterior stability index, middle/lateral stability index, and overall stability index) were evaluated.	Stretch-shortening cycle exercises performed on a trampoline for a period of 12 weeks are probably helpful for enhancing muscular strength and postural control in kids with Down syndrome and should be incorporated into their rehabilitative regimens.
17.	Michale O Ogundele 2023 [24]	Impact of non-pharmacological interventions on both the core symptoms of ADHD and the accompanying behaviors separately, as they are two distinct but equally significant problems.	The evaluation is conducted within the headings of Cognitive Behavioral Therapy, Neurofeedback, Psychoeducation, and Medication.	By the time they reach school age, 3% to 9% of children have ADHD, which frequently lasts into adulthood. It is the most prevalent developmental condition of childhood. For the affected children and young people, their careers, and society as a whole, ADHD in children and adolescents has far-reaching, multimodal effects.
18.	Ping Tao Tseng et al. 2018 [25]	The current study's aim was to conduct an updated systematic review and meta-analysis taking into account variations in peripheral iron levels and all other aspects of iron status between kids with ADHD and HCs. We also wanted to investigate the relationship between the risk and severity of ADHD.	The investigation adhered to the MOOSE recommendations.	There is growing recognition that iron deficiency, in particular, may raise a child's risk of attention-deficit hyperactivity disorder (ADHD).
19.	Carmen Giannantonio et al. 2010 [26]	The study focused on the effects of blood gases and oxygen saturation on spontaneous breathing by Vojta method	This study investigated the application of reflex rolling from the Vojta method in preterm children with lung illness.	The Vojta technique is safe for preterm neonates, according to our experience with it, but more research is required to confirm its positive effects and analyze long-term respiratory outcomes.
20.	Beeby et al. 1998 [27]	To quantify the frequency of thoracic musculoskeletal abnormalities and related elements preterm.	Infants in their first year of life who were born prematurely and weighed less than 2000 grams and were monitored at the preterm clinic from February 2007 to December 2008 participated in this cross-sectional study.	Preterm neonates frequently exhibited thoracic anomalies, which were associated with pulmonary disease and an inadequate length-to-age ratio.
21.	Abhishta P Bhandari 2022 [28]	To compare the effects of various body positions on hospitalized infants and kids with acute respiratory distress syndrome who are between the ages of four weeks and 16 years.	The concepts of oxygen saturation, partial pressure of arterial oxygen, oxygenation index, thoraco-abdominal synchrony, and episodes of desaturation were employed with the terms of supine positioning and prone positioning, respectively.	Despite the fact that the included studies suggest that the prone position may have some benefits, there was not enough information to offer specific recommendations. According to a preliminary study, positioning seems to improve oxygenation in children with ARDS who are mechanically ventilated.
22.	Ruth Van Der Looven et al. 2020 [29]	Current study aim is to present a thorough update on the most common and important risk factors for NBPP.	On the basis of the MOOSE recommendations and the PRISMA statement, a meta-analysis was carried out for the five most important risk factors. There were reported pooled odds ratios with 95% confidence intervals and study heterogeneity.	The incidence of neonatal brachial plexus palsy is declining. Shoulder dystocia, macrosomia, maternal diabetes, tool delivery, and breech birth all provide risks for neonatal brachial plexus palsy. The cesarean section appears to be a safety net.
23.	Jessica Anne Rich et al. 2019 [30]	BMJ case reports 2019 Dec 23	After undergoing surgery for a complex brachial plexus injury, the patient had six weeks of postoperative physical therapy.	Neuromuscular electrical stimulation can be used successfully as a rehabilitation adjunct to boost nerve regeneration after severe brachial plexus impairment. Physiotherapy therapies for brachial plexus and polytrauma injuries can help with strength, range of motion, and functional rehabilitation.

Discussion

Pediatric physical therapists give outstanding care to everyone, from infants in the neonatal intensive care unit to young adults with childhood illnesses. Worldwide, prematurity is the leading cause of neonatal deaths, and pneumonia is the second-leading cause of death among children under five. According to the World Health Organization (WHO), India has the world's highest rate of preterm deliveries (Table 4). Studies have shown that early intervention therapy and chest physiotherapy for neonatal intensive care unit secretion clearance that comprises manual hyperinflation and vibrations significantly reduce the preterm death ratio. Even children who are malnourished can benefit from physical therapy to build muscle while they are recovering. For children with cerebral palsy, the most cutting-edge treatment, Pedi suit/Rehab suit therapy, is required. The benefits of multimodal stimulation for preterm infants include promoting eating and psychomotor growth. The multimodal stimulus has also been shown to enhance visual function. The General Movement Assessment approach is utilized for baby neurodevelopment. It is a method that aids in the early diagnosis of cerebral palsy. The Albert Infant Motor Scale and Optimality Score are two components of the General Movement Assessment. The handling techniques are especially useful in respiratory disorders like the incidence of intermittent hypoxemia, where it is stated that touch features are basic trigger mechanisms for intermittent hypoxemia [31-33].

**Table 4:** Illustration of treatment Refs. [1-35].

Treatment	Elucidation
Multimodal stimulus	Multimodal stimulus is a term used in pediatric physical therapy to describe the utilization of numerous sensory inputs and stimuli to activate many sensory modalities at once during therapeutic interventions. To promote and facilitate a child's motor, cognitive, and sensory development, these stimuli are used. Therapists strive to provide a comprehensive and dynamic environment that fosters holistic growth and increases the efficiency of therapy for children by combining different sensory experiences, such as visual, auditory, tactile, proprioceptive, and vestibular inputs. Based on the knowledge that sensory experiences are essential to a child's growth and learning, multimodal stimulation is used in pediatric physical therapy. Therapists can improve a child's sensory integration, motor coordination, cognitive ability, and general well-being by using a variety of sensory inputs. Multimodal stimulation approaches can entail a variety of exercises, such as interactive games, virtual reality simulations, music-based exercises, sensory integration therapy, and the use of adapted equipment, all of which can be specifically tailored to match the needs and objectives of each child receiving therapy. Multimodal stimulus is being used in pediatric physical therapy with the overall goal of offering a thorough and interesting therapeutic experience that helps kids meet developmental milestones, develop their motor skills, encourage sensory processing, and improve their general quality of life.
Handling techniques	Pediatric physiotherapy handling skills are crucial because they create the foundation for encouraging children's healthy motor development, sensory integration, and general well-being. In addition to improving muscle strength, joint mobility, and postural control, skilled handling is essential for avoiding deformities, contractures, and other issues. Physiotherapists can support active movement participation, encourage neurodevelopmental advancement, and foster positive caregiver-child interactions during therapeutic interventions. This will help children feel secure and emotionally connected. To enhance children's quality of life, ensure their holistic development, and unlock their full potential for independence and useful living, effective handling skills are essential.
Stretching	Stretching is a crucial component of pediatric physical therapy because it helps children increase their flexibility, mobility, and overall musculoskeletal health. Physiotherapists can assist in enhancing posture, joint range of motion, and muscle suppleness by introducing suitable stretching exercises into therapy sessions. This helps to avoid the onset of musculoskeletal impairments and promotes optimal physical function. Additionally, stretching helps lower the likelihood of muscular tightness, contractures, and joint stiffness, especially in children with neurological disorders or those who have been immobile for an extended length of time. Additionally, physiotherapists can foster healthy behaviors that support the maintenance of musculoskeletal health, boosting long-term physical well-being and improving the overall quality of life for pediatric patients by encouraging frequent stretching exercises.
Microcurrent therapy	Microcurrent therapy plays a significant role in pediatric physical therapy because it offers a non-invasive and gentle way to help kids with their pain, increase tissue healing, and improve their overall recovery. Microcurrent therapy can effectively target particular areas of pain and inflammation, promoting cellular regeneration and accelerating the healing process without causing discomfort or unfavorable side effects. It works by using low-level electrical currents that mimic the body's natural bioelectrical signals. Children who have musculoskeletal injuries, neurological disorders, or chronic pain can benefit most from this therapy since it lessens pain perception, enhances local blood circulation, and encourages the creation of endogenous neurotransmitters and cellular healing processes. Pediatric physiotherapists can offer a safe and efficient method to enhance the rehabilitation process, hasten healing, and more by including microcurrent therapy in treatment programs.
Vojta approach	Pediatric physical therapy places a high value on Vojta treatment because it provides a specific and all-encompassing method for promoting children's postural control, movement patterns, and general motor development, especially in those with neuromuscular abnormalities or developmental delays. Vojta therapy uses precise pressure techniques on critical reflex zones to elicit involuntary motor responses, encouraging deep muscle activation and facilitating coordinated movement patterns necessary for reaching developmental milestones. The goal of this therapy is to stimulate and rewire the central nervous system, enhancing postural stability, balance, and motor performance. Additionally, Vojta treatment is critical for fostering the growth of core motor skills, increasing the child's independence, and facilitating the integration of basic movement patterns, all of which eventually contribute to the child's total physical and functional well-being.
Neurodevelopmental therapy	Pediatric physical therapy places a great deal of emphasis on neurodevelopmental treatment (NDT), which offers a thorough and research-based strategy to encourage motor learning, functional independence, and ideal movement patterns in kids with neurological impairments or developmental disabilities. NDT seeks to promote the integration of typical movement patterns and postural responses by addressing the underlying neurophysiological principles, hence promoting the development of motor control, balance, and coordination. To foster active engagement and the development of practical skills necessary for daily living, this method places an emphasis on the use of guided handling techniques, therapeutic exercises, and task-oriented activities. Pediatric physiotherapists can assist children in enhancing their motor function, independence, and capacity for involvement in a variety of activities by the use of NDT principles, ultimately leading to an improvement in quality of life.
Proprioceptive and visual training	In pediatric physical therapy, proprioceptive and visual exercises are essential because they improve children's motor coordination, sensory integration, and overall functional performance. Physiotherapists can aid in the development of body awareness, spatial orientation, and postural control, supporting enhanced balance and stability during a variety of motor tasks, by adding particular exercises and activities that target proprioceptive input and visual-motor coordination. These training methods help improve sensory-motor integration and encourage the effective use of visual information in movement planning and execution, which is especially helpful for kids with sensory processing issues, developmental coordination issues, or neurological impairments. Physiotherapists can assist kids in acquiring crucial motor skills, enhancing their coordination, and improving their general ability by including proprioceptive and visual training in their therapy sessions.

Deformities involving muscles, tissue, or joints are included in musculoskeletal conditions. Exercises for stretching, handling placement, and strengthening are used to treat this issue [34]. Even adjustments to the surroundings help treat the illnesses. Higher-level data are emerging in favor of microcurrent therapy for torticollis. As the cesarean section appears to be a precaution, Erb's palsy, also known as neonatal brachial plexus palsy, is decreasing. Physiotherapy therapies are useful in assisting and enhancing the range of motion, strength, and functional recovery after brachial plexus and polytrauma injuries. One of the cardiorespiratory illnesses is acute respiratory disorder syndrome (ARDS). In premature neonates, chest physical therapy can treat lung issues. The Vojta approach is useful for treating lung problems in chest physical therapy. Both prone and supine positions were used while measuring oxygen saturation, partial pressure of arterial oxygen, oxygenation index, and thoracoabdominal synchronization [35].

Children with cerebral palsy are offered stretches and progressive resistance exercises to maintain and build strength. The core stability activities also improve children's balance and coordination. Patients with cerebral palsy and feeding issues might use the Bobath techniques for neurodevelopmental therapy to help with feeding and swallowing tasks. The spastic diplegic condition that children experience in cerebral palsy may be supported by simultaneous proprioceptive and visual training, but it has little or no impact on kinetic gait characteristics. Stretch-shortening cycle exercises based on the trampoline are used to address children's postural control issues as well as to improve strength in children with Down syndrome. Pediatric physiotherapists have a bright future in the rehabilitation of young athletes. Depending on the child's needs, a therapist will undertake sports conditioning, which may involve balance, proprioception, agility, coordination training, postural and gait retraining, dynamic core strengthening, and flexibility training. This is done to reduce the likelihood of sports-related injuries [12].

Limitations

The limitation of the topic presents a difficulty in fully encompassing the broad range of current pediatric physical therapy approaches and their significance within the context of this review. The overview may not adequately cover all the specific methods, case studies, or clinical applications needed for a complete grasp of pediatric physical therapy practices because of being very condensed.

Furthermore, given that these factors can have a substantial impact on the execution and efficacy of the interventions, the brief summary might not fully address regional or cultural variations in pediatric physical therapy practices. The review does not take into consideration the variations in access to pediatric physical therapy treatments and different socioeconomic backgrounds, which could restrict the generalizability of the findings to all populations and communities.

Additionally, the review might not go into great detail on the difficulties and barriers that pediatric physical therapists encounter in actual clinical settings, such as resource limitations, staffing problems, and changing healthcare regulations. These difficulties, which are so important in determining how pediatric physical therapy services are actually provided, might not be adequately covered in a succinct description. The succinct summary can also miss some important trends and future developments in pediatric physical therapy, such as the potential effects of new interdisciplinary collaborations, technological innovations, and scientific breakthroughs. These factors are essential for comprehending the changing environment of pediatric physical therapy and assuring its ongoing expansion and efficacy. As a result, while providing an insightful introduction, the succinct overview might not fully capture the breadth and depth of the topic of pediatric physical therapy, leaving room for further exploration and analysis.

## Conclusions

This study focuses on the current situation of physiotherapy techniques for resolving and enhancing the issues that are developing in pediatric patients. In conclusion, pediatric diseases like spina bifida, congenital torticollis, and cerebral palsy can be managed and improved with physiotherapy. The in-depth investigation reveals the widespread usage of strengthening and stretching workouts for condition control. Beyond correcting errors, it encourages children's physical, cognitive, emotional, and social development, enabling them to realize their full potential. Additionally, the use of cutting-edge methods and technology has improved pediatric physical therapy's effectiveness and engagement levels, improving the experience for patients as a whole. A child's sense of independence and self-confidence are also fostered by therapists in addition to physical disabilities. The importance of pediatric physical therapy must be acknowledged and supported by healthcare professionals, carers, and legislators as the discipline develops. By doing this, we can work together to give physically challenged children a better future, so that they can have healthier, more contented lives. Pediatric physical therapy is a glimmer of hope and advancement for the most impressionable individuals of our society in this constantly changing environment.
